# Challenges in the Development of Drug Delivery Systems Based on Small Extracellular Vesicles for Therapy of Brain Diseases

**DOI:** 10.3389/fphar.2022.839790

**Published:** 2022-03-29

**Authors:** Gecioni Loch-Neckel, Ana Teresa Matos, Ana Rita Vaz, Dora Brites

**Affiliations:** ^1^ Neuroinflammation, Signaling and Neuroregeneration Lab, Research Institute for Medicines (iMed.ULisboa), Faculty of Pharmacy, Universidade de Lisboa, Lisbon, Portugal; ^2^ Department of Pharmaceutical Sciences and Medicines, Faculty of Pharmacy, Universidade de Lisboa, Lisbon, Portugal

**Keywords:** drug delivery systems, microRNA nanocarriers, biomarkers, neurodegenerative diseases, brain tumors, cargo of sEVs/exosomes, isolation and loading of sEVs/exosomes

## Abstract

Small extracellular vesicles (sEVs) have ∼30–200 nm diameter size and may act as carriers of different cargoes, depending on the cell of origin or on the physiological/pathological condition. As endogenous nanovesicles, sEVs are important in intercellular communication and have many of the desirable features of an ideal drug delivery system. sEVs are naturally biocompatible, with superior targeting capability, safety profile, nanometric size, and can be loaded with both lipophilic and hydrophilic agents. Because of their biochemical and physical properties, sEVs are considered a promising strategy over other delivery vehicles in the central nervous system (CNS) since they freely cross the blood-brain barrier and they can be directed to specific nerve cells, potentiating a more precise targeting of their cargo. In addition, sEVs remain stable in the peripheral circulation, making them attractive nanocarrier systems to promote neuroregeneration. This review focuses on the recent progress in methods for manufacturing, isolating, and engineering sEVs that can be used as a therapeutic strategy to overcome neurodegeneration associated with pathologies of the CNS, with particular emphasis on Alzheimer’s, Parkinson’s, and amyotrophic lateral sclerosis diseases, as well as on brain tumors.

## 1 Introduction

**FIGURE 1:**
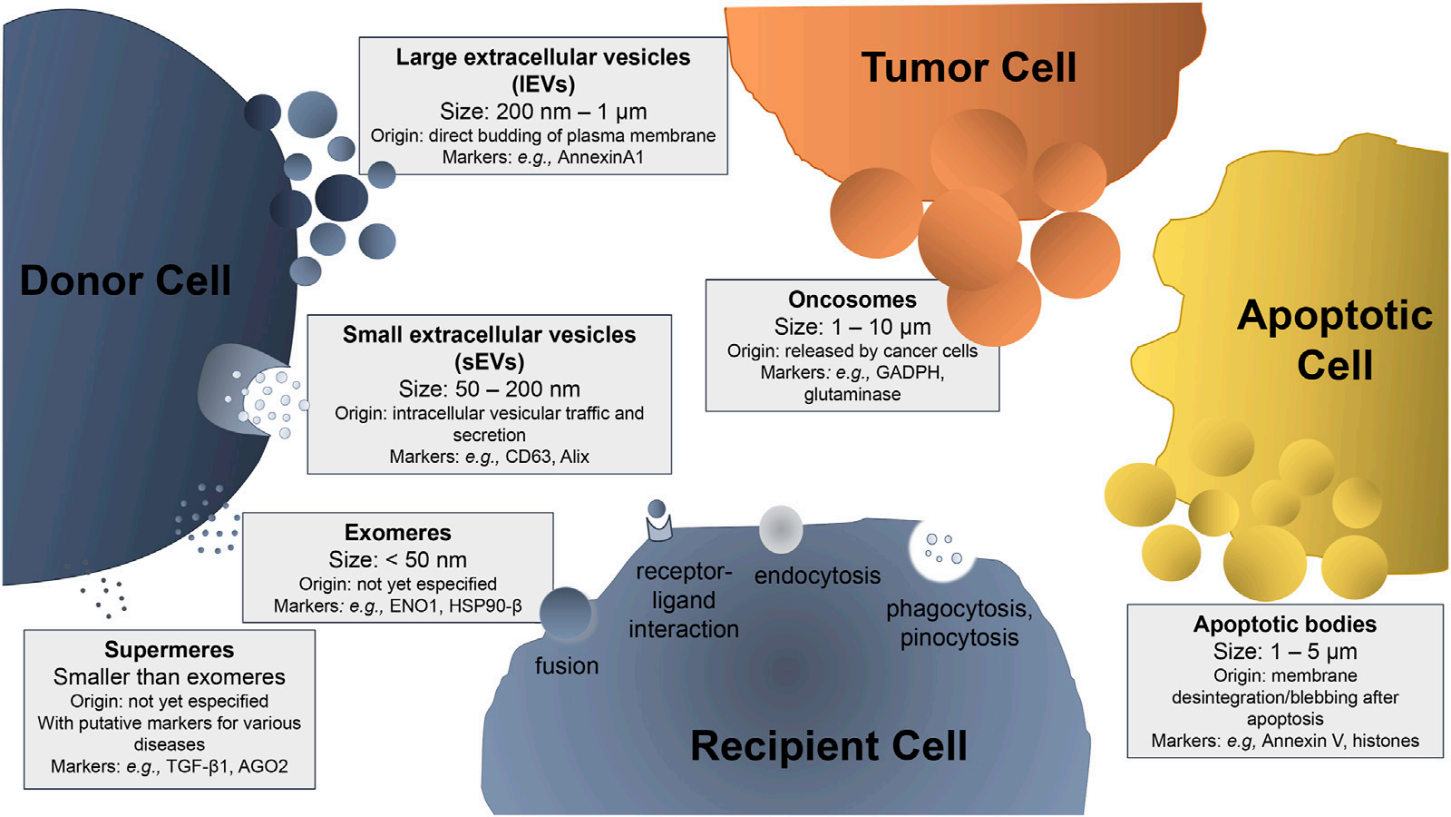
Schematic representation of the extracellular vesicles (EVs) subtypes, sizes, and characteristic markers. Depending on their size and site of origin, EVs can be classified as: i) large extracellular vesicles (lEVs), also mentioned as ectosomes or microvesicles, when their size ranges from 200 nm to 1 μm, generated from the budding of the plasma membrane; ii) small extracellular vesicles (sEVs), also referred as exosomes, with a diameter from 50 to 200 nm, which are formed after inward budding of endosomal vesicle membrane and maturation in multivesicular bodies that later fuse with the plasma membrane to be secreted into the extracellular space; iii) oncosomes, which are EVs secreted by tumor cells, with a size ranging from 1 to 10 μm (relatively larger than lEVs) and responsible for the spreading of the tumor; iv) apoptotic bodies that are EVs secreted by apoptotic cells upon their membrane disintegration after apoptosis, with a size ranging from 1 to 5 μm; (v) amembraneous exomeres that present a size smaller than 50 nm, whose origin is not fully understood yet; and (vi) amembraneous supermeres that are smaller and morphologically distinct from exomeres. The cell that releases EVs into the medium is called the donor cell and the one that internalizes EVs is the recipient cell. EV internalization in the recipient cell can occur by dissecting mechanisms, such as: a) fusion, when the membrane of the vesicle becomes contiguous with the cell membrane, releasing its contents into the cell; b) receptor-ligand interaction, when the vesicle has a specific ligand in its membrane that will bind to a specific receptor on the cell membrane allowing its internalization; c) endocytosis, when the vesicle is internalized by the plasma membrane; d) phagocytosis, when the vesicle is larger than 0.5 μm, being engulfed by the target cell; and e) pinocytosis in the case of fluid absorption (macropinocytosis of solute molecules larger than 200 nm and micropinocytosis of smaller particles). Some examples of characteristic markers used in EV identification are included.

The term “extracellular vesicles” refers to particles naturally released from the cell that do not contain a functional nucleus, i.e., they cannot replicate themselves (Johnstone, 2005). Extracellular vesicles (EVs) are released by most cell types and can be classified into different subtypes, including large and small extracellular vesicles (respectively, lEVs and sEVs), exomeres, supermeres, oncosomes, and apoptotic bodies (El Andaloussi et al., 2013; Brites, 2020; Zhang et al., 2021). These EVs are different in size, density, biochemical and biophysical properties, as well as in secretion pathways, which may depend on the donor cells that produce them (Figure 1). EVs can be taken up by the recipient cells through different mechanisms, including phagocytosis, micropinocytosis, receptor-ligand interaction, or membrane fusion.

Apoptotic bodies result from the fractionation/fragmentation of the cellular content of cells that die by apoptosis. These bodies are formed during membrane disintegration by a separation of the plasma membrane from the cytoskeleton (Jiang and Poon, 2019). Apoptotic bodies are quite variable in size and cargo. They have a size ranging from 1 to 5 µm and their cellular contents includes intact organelles, with high levels of proteins associated with the nucleus, chromatin residues, DNA fragments, RNA (in a large amount), degraded proteins, organelles fragments, and glycosylated proteins (in a small amount) (Xu et al., 2019; Battistelli and Falcieri, 2020).

lEVs, also referred to in the literature as ectosomes or microvesicles (MVs), are originated from the cellular membrane through budding and fission. After their generation, lEVs are released within the extracellular space, enter in the circulation, and transfer their cargo to either neighboring or more distant cells (Akers et al., 2013). lEVs are quite heterogeneous in size, ranging from ∼200 nm to more than 1–2 μm in diameter. Their cargo usually reflects both the intracellular origin and the cell type from which they are derived, and may contain cytoskeletal proteins, heat shock proteins, integrins, nucleic acids, bioactive lipids, and other active components expressed by the cells of origin (Lv et al., 2019). Some markers used in their characterization are included in Figure 1, though their specific characterization is not trivial (Phan et al., 2021) and some of the markers despite being more abundant in lEVs can be also found in sEVs and vice-versa (Théry et al., 2018; Saludas et al., 2022).

sEVS, also referred to as exosomes, derive from the endosome pathway. They have tightly controlled biogenesis and regulated secretion into the extracellular media. sEVs are typically 30–200 nm in diameter and are the most homogeneous (in both shape and size) population of extracellular vesicles (Yuyama and Igarashi, 2016). They are lipid bilayer bound vesicles that are easily uptaken by the mononuclear phagocyte system, allowing them to reach other cellular targets beyond the ones from which they derive (Antimisiaris et al., 2018). sEVs are constitutively or stimulus-dependently secreted from many different cell types, including those of the nervous system, such as neurons, astrocytes, oligodendrocytes, and microglia (Caruso Bavisotto et al., 2019; Song Z. et al., 2020). sEVs are important mediators in cell-to-cell and inter-tissue communication, by carrying small noncoding ribonucleic acids (ncRNAs), messenger RNAs (mRNAs), lipid molecules, and proteins. sEVs play critical roles in regulating both physiological and pathological processes. Indeed, in pathological conditions, the cargo transferred by sEVs may have detrimental effects, while contributing for the spread of the disease, which has been described in inflammation-associated and neurodegenerative diseases, as well as in tumor growth (Johnstone, 2005; Ciregia et al., 2017; Isola and Chen, 2017; Busatto et al., 2021). Recent evidence indicates that sEVs released by the different tissues can be collected from body fluids, in order to evaluate their unique protein or RNA content to be used as disease biomarkers, or as therapeutic tools in different pathologies (Barile and Vassalli, 2017). Some examples were already described in the cancer field, where the tumor-derived sEVs were demonstrated to be enriched in certain miRNAs that could act as tumor markers (Kumar et al., 2015), or in circulating sEVs derived from glioblastoma patients, which have showed increased levels of Epidermal Growth Factor Receptor (EGFR)-vIII mRNA (Skog et al., 2008). Other examples related with the central nervous system (CNS) disorders include the AT270 phospho-tau, a biomarker for Alzheimer’s disease (AD), detected in sEVs collected from the cerebrospinal fluid (CSF) of patients with mild disease (Saman et al., 2012), and syntenin one that was found elevated in the circulating sEVs isolated from the serum of Parkinson’s disease (PD) patients (Tomlinson et al., 2015). Several studies using different body fluids and their isolated sEVs propose them as potential candidates for early diagnosis in neurodegenerative diseases based on the disease-associated mutant proteins and miRNAs (Hornung et al., 2020; Rastogi et al., 2021). However, the majority of the studies are retrospective with incomplete clinical and pathological informations (Wong and Chen, 2019). Moreover, though the quick and accurate isolation of sEVs is key for their application, current methods still have limitations, such as time-consuming processes, presence of contaminants, and high costs (Xiao et al., 2020). Standard isolation protocols are not yet established and sEVs usually represent a heterogenous population derived from different cell sources. Lately, separation of sEVs originated from neurons, microglia and astrocytes were achieved by using specific cell surface markers and magnetic beads (Kumar et al., 2021).

Another type of EVs referred to as oncosomes are atypical EVs derived from cancer cells, with larger sizes (1–10 µm), which may carry abnormal macromolecules including oncoproteins. They are produced from non-apoptotic plasma membrane blebbing from cancer cells and can mediate the communication between cancer and non-cancer cells within the tumor microenvironment (Jaiswal and Sedger, 2019).

In recent years, a novel population of EVs smaller than sEVs (<30 nm of diameter) that can be isolated from the sEVs by an ultracentrifugation-based method was described (Zhang Q. et al., 2019). Although their function is still a matter of debate, it is known that exomeres are enriched in proteins involved in cellular bioenergetics, namely in glycolysis and Mechanistic Target of Rapamycin Complex 1 (mTORC1) metabolic pathways, suggesting their potential association with mitochondrial function. In the same study, the authors demonstrated that exomeres are enriched in proteins associated with the endoplasmic reticulum, mitochondria, and microtubules, suggesting that these proteins may be implicated in their biogenesis or even secretion. Nucleic acids and lipids are also reported as part of exomeres’ cargo. Lately, supermeres, nanoparticles smaller than sEVs and exomeres, and morphologically distinct from exomeres, were described to be easily ingested and enriched with cargo involved in several cancers, as well as Alzheimer’s and cardiovascular diseases (Zhang et al., 2021). They are enriched in proteins and miRNAs, as well as miRNA-processing proteins such as Argonaute RISC Catalytic Component 2 (AGO2), and are functional agents of intercellular communication, also constituting candidate biomarkers and therapeutic targets (Clancy et al., 2021).

Such heterogeneity of EVs needs further attention to understand the nature of each subpopulation in a more specific manner. In this review we recapitulate recent progress in methods for manufacturing, isolating, and engineering sEVs, and how they can be used to treat brain diseases. As endogenous nanovesicles, sEVs also have many of the desirable features of a good drug delivery system. They are naturally biocompatible, with superior targeting capability, safety profile, nanometric size, and can be loaded with both lipophilic and hydrophilic agents. Because of these highly desirable properties and their ability to penetrate biological barriers, sEVs represent ideal natural nanocarriers for the treatment of brain diseases (Busatto et al., 2021; Elliott and He, 2021). In terms of their transport and properties, sEVs are promising carrier vehicles for the transfer of drugs across the blood–brain barrier (BBB).

## 2 Biogenesis, Production, and Storage of sEVs

Due to the growing number of studies using EVs in the last years, there was a need to create guidelines for the standardization of protocols of separation and characterization, nomenclature, and usage of the different types of EVs. Guidelines were part of the first Minimal Information for Studies of Extracellular Vesicles (MISEV) document, which was released in 2014 by the International Society for Extracellular Vesicles (ISEV). More recently, MISEV2018 guidelines were published in the *Journal of Extracellular Vesicles* (Théry et al., 2018). The purpose of this document was to provide an overview of the recommended procedures among the standardized methods in EV research. The information related with EV biogenesis, uptake, and signaling is more consensual, although storage and stability issues remain a matter of discussion among the scientific community, as well as the processes of EV fusion with the target cells (Russell et al., 2019). In this chapter, we will summarize the current knowledge on biogenesis, production, and storage of sEVs.

### 2.1 Origin

**FIGURE 2:**
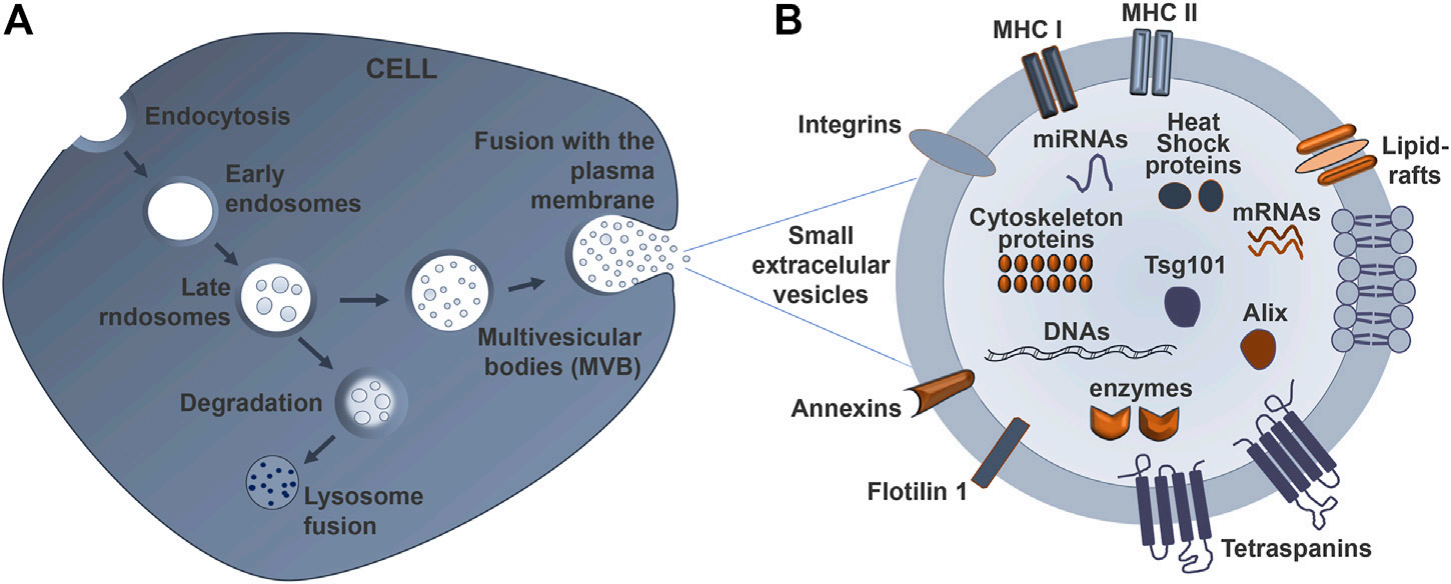
Schematic representation of sEV biogenesis and its typical structure. **(A)** Early endosomes mature into late endosomes named multivesicular bodies (MVBs), which are formed after inward budding of the plasma membrane. The MVBs can either fuse with lysosomes to degrade their cargo, or fuse with the cell membrane to release small extracellular vesicles (sEVs) into the extracellular space, thus mediating cell-to-cell communication. **(B)** The sEV is limited by a lipid bilayer that includes ceramide sphingolipids and phospholipids. The sEV membrane also contains various proteins involved in the antigen presentation (major histocompatibility complexes–MHC I and II), as well as targeting and adhesion (integrin and tetraspanins) proteins, together with annexins. The cytosol content of the sEVs varies according to the cell and tissue from which they derive, and may contain lipids, nucleic acids, and proteins, among other components.

sEVs are secreted by all cells and are originated as intraluminal vesicles during the process of multivesicular body formation. The biogenesis of sEVs has been addressed by many publications (Baumann, 2021; Brites, 2020; Russell et al., 2019; van Niel et al., 2018). Briefly, the biogenesis of sEVs (Figure 2A) consists of three different stages: (1) the formation of endocytic vesicles from the plasma membrane, (2) the inward budding of the endosomal vesicle membrane that maturates in multivesicular bodies (MVBs), which consist of intraluminal vesicles (ILVs), and (3) the fusion of MVBs with the plasma membrane (instead of being sent to degradation into the lysosomes), allowing the release of these ILVs, including sEVs, into the extracellular space (Zhang Y. et al., 2019). The pattern of nanospherical membrane-type is derived from the parent cells from which the sEVs are formed. Their respective cargo will likewise vary depending on the cell type of origin and status (Kang et al., 2021). The sEV membrane is formed by two layers of proteins and lipids, including cholesterol, phospholipids, glycerophospholipids, and sphingolipids that maintain its stability and structure (Figure 2B). Their lipid composition accounts for their unique rigidity (Skotland et al., 2019; Lin Y. et al., 2020).

**FIGURE 3:**
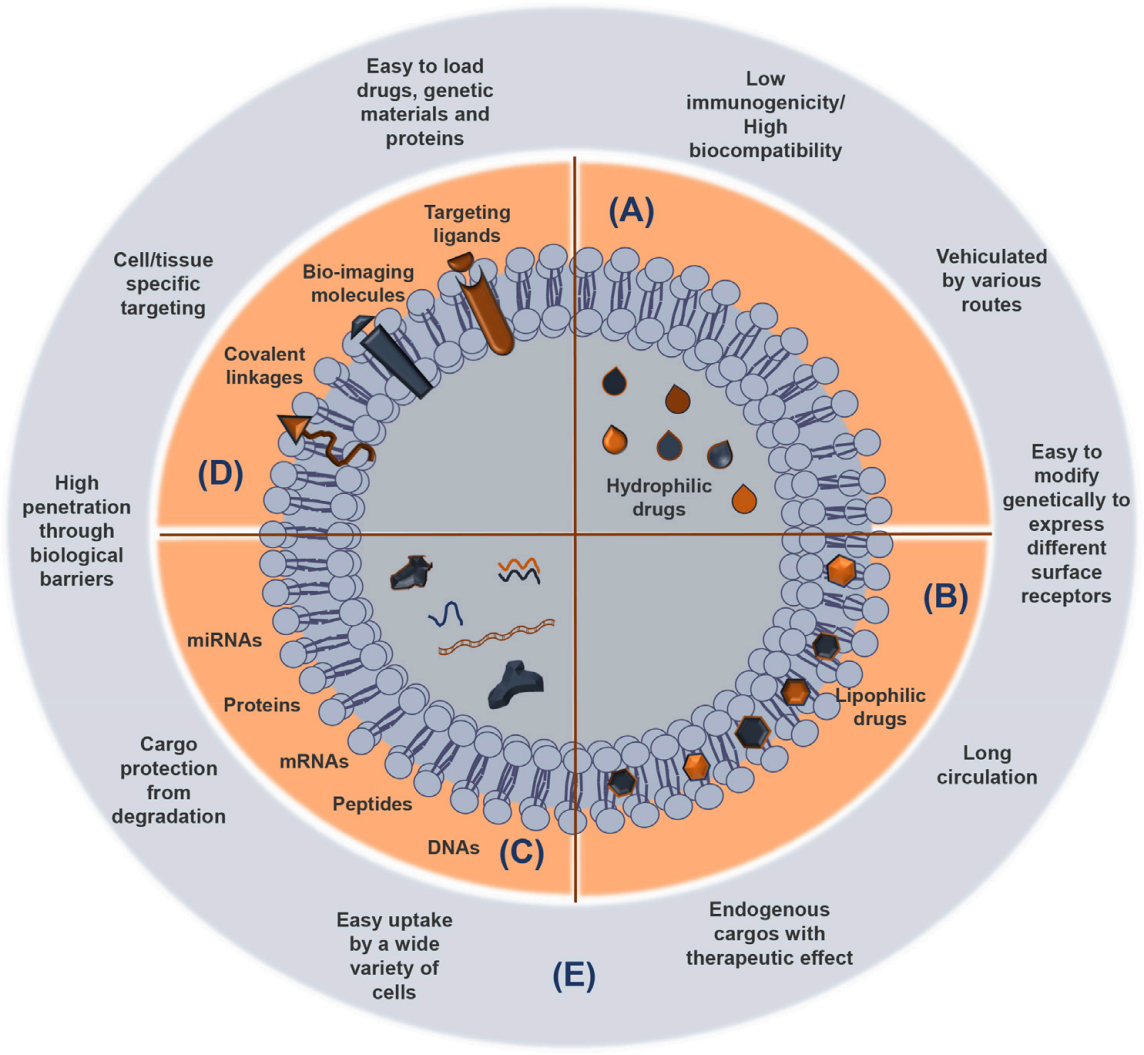
sEV cargoes and advantages as delivery systems of functional and therapeutic molecules. Small extracellular vesicles (sEVs) consist of an aqueous compartment surrounded by a lipid bilayer. sEVs can compartmentalize and solubilize hydrophilic compounds in the aqueous compartments **(A)** and lipophilic molecules almost totally entrapped in the lipid layer **(B)**, which protects them from degradation. Agents with intermediary partition coefficient are equally distributed between the aqueous and the lipid compartments. Protein, peptides, and genetic material can be also released from sEVs **(C)**. Attachment of targeting compounds, bio-imaging molecules, and covalent linkage to sEV surface contribute to enhance their utility as vehicles to deliver biomolecules and drugs **(D)**. The most important advantages of the sEVs as therapeutic nanocarriers are indicated **(E)**.

After sEV release by exocytosis, their cargo is protected from enzymes like proteases and ribonucleases by their lipid bilayer membrane (Lorenc et al., 2020) (Figure 3). Once at the extracellular space, sEVs are transferred into the recipient cells through interaction with proteins that facilitate subsequent endocytosis by specific processes, such as receptor interaction, membrane fusion, and internalization. The internalization step of sEVs by the recipient cells can occur via receptor-ligand interactions, direct fusion of membranes, or internalization via endocytosis, and is normally dependent on the cell type, as recently reviewed by Malloci and others (Malloci et al., 2019). For example, the selective transfer of sEVs from oligodendrocytes and their subsequent uptake by microglia through a macropinocytosis mechanism does not require binding to the specific receptor (Fitzner et al., 2011). Another example is the injection of oligodendroglia sEVs in mouse brain, which results in a functional retrieval of sEV cargo in neurons through a clathrin-dependent endocytosis (Fruhbeis et al., 2013). Another study refers to the release of EVs from primary cortical astrocytes and microglial cells being triggered by ATP-mediated activation of P2X7 receptors after contact with phosphatidylserine at the cell surface (Bianco et al., 2009). sEVs carrying a multitude of proteins, such as myelin proteins, as well as RNA, are released from oligodendrocytes and endocytosed by neurons (Frohlich et al., 2014). In addition, the release of serotonin from neurons has been implicated in the release of microglial sEVs, upon binding of serotonin to specific receptors found in the microglia, suggesting a neurotransmitter dependent release (Glebov et al., 2015). To sum up, sEV uptake is determined by multiple mechanisms, together with cell-dependent different combinations of strategies. These specificities should be considered when designing sEV-based therapies.

### 2.2 Source

sEVs exert unique biological activities by stimulating regeneration and modulating pathological conditions, properties that can be explored for medicinal purposes. Namely, they are able to transfer RNA and proteins from donor cells to other cells in the surrounding milieu (Simons and Raposo, 2009). Furthermore, EVs derived from mesenchymal stem cells (MSCs) appear particularly useful in enhancing recovery from various injuries. MSCs are commonly used as a source of sEVs because they can recapitulate the biological activity of MSCs and have been used as a cell-free therapeutic (Harrell et al., 2021). For example, stem cells may modulate their biological effects through the delivery of genetic information that will alter the gene expression of the target cells (Zhao et al., 2019). In addition, EVs from both immune and non-immune cells are shown to suppress or stimulate both adaptive and innate immunity, which effects likely depend on the environmental context, as well as on the type of EV from a particular immune cell (Kim et al., 2005; Bu et al., 2015). sEVs are potential fingerprints of their originating cells and their composition largely depends on the donar cell, although it can also be determined by the cell metabolism and its needs, as well as be influenced by cellular and environmental factors (Bell and Taylor, 2017). As an example, release of miRNAs from cells into sEVs can occur either passively or actively. Therefore, it is very important to choose the cell lines that will act as the source of sEVs, once the donor cell will determine their ability to selectively target cells (Sancho-Albero et al., 2019). It has been shown that the interaction between the drug delivery systems is affected by the EV surface proteins (Yin et al., 2013). The ability of different types of EVs to modulate immune responses allows their therapeutic potential as a tool with theragnostic applications in nanomedicine (Thomas et al., 2021). Therefore, we may enrich sEVs with promising curative molecules or engineering cell lines to produce sEVs with a specific and desired target specificity (Gao et al., 2018).

### 2.3 Strategies to Enhance Production

The use of sEV technology as drug delivery vehicles must meet two basic requirements: first, a reproducible and scalable isolation with established production protocols to achieve high purity and elevated yield of a defined population of sEVs for clinical use; and second, the high drug-loading content in sEVs must be enough to obtain a therapeutic response (Armstrong and Stevens, 2018; Lin Y. et al., 2020; Wu Z. et al., 2021). Cells may produce higher or lower number of sEVs depending on several factors, but what will be important is that the production of enormous quantities of vesicles does not modify cell characteristics and behavior. Alterations in the composition and function of the sEVs may be performed using environmental parameters, such as pH gradients, mass transfer, or hydrodynamic force during scale-up processes. The number of sEVs released depends on the cell type, physiological state, and microenvironmental conditions. The production increases in cancer cells under hypoxic conditions, in melanoma cells under acidic microenvironment, and under cell stress induction with calcium ionophores (Zaborowski et al., 2015). It was indicated that immature dendritic cells produce a limited number of sEVs in the µg range (Alvarez-Erviti et al., 2011). However, MSCs secrete more elevated amounts, usually in the milligram range (Chen et al., 2011), making them a more relevant source for the production of therapeutic EVs targeting neurodegenerative diseases. Nevertheless, it should be taken into account that MSCs may undergo senescence after a few passages, a condition that will lead to the production of sEVs with impaired regenerative capacity as compared to those from younger cells (Severino et al., 2013). The confluence of cell cultures also plays a critical role in the biogenesis and secretion of sEVs. Cell culture in a pre-confluent state showed a decreased sEV secretion compared to the confluent one, potentially due to changes in cholesterol metabolism (Llorente et al., 2013). In addition, sEV secretion rate from the 3D cultured cells is better when cells are close to confluence (Thippabhotla et al., 2019).

Contact inhibition between cells can also reduce sEV release, since these cells become quiescent and do not actively divide (Whitford and Guterstam, 2019). Apart from the individual characteristics of the cell culture and of the cell type that originates the sEVs, some methods are designed to increase their total yield, such as intracellular calcium production (Savina et al., 2003), external stress (de Jong et al., 2012), cytoskeletal blocking (Parolini et al., 2009), drug stimulation (Datta et al., 2018; Wang et al., 2020), and induction of gene expression factors (Boker et al., 2018). In spite of increasing the sEV yield, such methods should be considered with caution, since they may also alter sEV composition, therapeutic efficacy, and safety (Emam et al., 2018; Whitford and Guterstam, 2019). Another aspect to take into consideration is the increased risk of contamination with apoptotic bodies (Emam et al., 2018; Whitford and Guterstam, 2019), although their presence was referred to also have important regulatory roles, and can be a mechanism to immunomodulate dying cells (Caruso and Poon, 2018).

Some recent studies report the relevance of neural cell-derived sEVs to be used in neurodegenerative conditions (Huo et al., 2021). The direct conversion of somatic cells into induced neural precursor cells (iNPCs) provides abundant production of sEVs (Ma et al., 2019; Yuan P. et al., 2021), mainly if using bioreactors (Baghbaderani et al., 2011; Miranda et al., 2016). These sEVs after transplantation revealed to have therapeutic properties in ischemic stroke (Zhang et al., 2020). Lately, studies considered that milk is a viable and safe source of sEVs for therapeutic delivery. For instance, breast milk was shown to contain EVs in large quantities and with an enriched cargo in miRNAs (Jiang et al., 2021). Moreover, it has been demonstrated that milk sEVs improve oral drug bioavailability by protecting their cargo against degradation by low pH, RNases, or conditions that mimic digestion in the gastrointestinal tract (Izumi et al., 2012). Other biofluids, such as saliva, blood, and urine, were also demonstrated to be an alternative source of sEVs. Nevertheless, each body fluid has a clearly distinct vesicle profile and it is not clear what is the delivery potential and safety of these systems for continuous or long-term continuous exposure (Yuana et al., 2013).

### 2.4 Large-Scale Production

Applying the sEV-based therapies to clinics has been slowed down due to sEV low availability and difficulties for large-scale production. Beyond the use of small molecule modulators capable of enhancing sEV secretion, e.g., norepinephrine, N-methyldopamine, and forskolin recently described for MSCs (Wang et al., 2020), other upscaling approaches and technologies for their production have been reported, assuring not only high quality but also high quantity (Andriolo et al., 2018; Mendt et al., 2018). These strategies involve standardized production methods that increase the volume of cell culture from flasks to containers, bioreactors, or hollow fibers, aiming production toward a good manufacturing practice–grade (GMP-grade) of sEVs. Different methods have allowed researchers to isolate and characterize sEV populations in a more accurate way. First works describing methods for the production, purification, and characterization of clinical grade sEVs (cGMP) emerged in the beginning of 2000 (Lamparski et al., 2002; Navabi et al., 2005).

Bioreactors and hollow-fiber perfusion bioreactors have been used in cell culture systems for a long time and they improve sEV yield. These reactors support large numbers of cells at high densities in a continuous mode without the need of splitting or subculturing these cells (Yamashita et al., 2018; Yoo et al., 2018). Using a hollow fiber bioreactor, Watson and others showed that the yield of sEVs obtained from the conditioned media of human embryonic kidney 239 cells (HEK293 cells) was 100 × 10^7^ particles per µg EV protein, instead of 20 × 10^7^ by the conventional flask culture (Watson et al., 2016). In another study with MSCs, the authors showed that once compared to the conventional methods, the bioreactor led to a 5.7-fold increase of EV concentration in the conditioned medium, with a threefold increase in the number generated per cell (MSC from bone marrow originated an average of 3 × 10^11^ EV particles per 60 ml final volume) (de Almeida Fuzeta et al., 2020). Additional advantages include less risk of contamination and less labor, besides the higher yield in a short incubation period. However, ideal criteria for production of large-scale GMP-grade EVs, such as scalability, reproducibility, size distribution, safety, surface charge, and purity of the resulting product still remain to be determined (Yamashita et al., 2018). Nevertheless, biomanufacturing of cells engineered to produce therapeutic proteins is well established, and practices or lessons from this area of industry could be transferred into the production of sEVs to be used in biomedicine.

### 2.5 Storage Conditions

Stability of products is mandatory and required for biomedical applications, such as the case of drug delivery systems. There are few research groups trying to understand the relationship between storage conditions and the integrity of sEVs (Jeyaram and Jay, 2017). The ISEV recommends that sEVs must be suspended in phosphate buffered saline (PBS) and stored at −80°C (Witwer et al., 2013). This recommendation was based on previous works showing that the structural and biological stability of the sEVs stored at −80°C for periods of up to 5 months have not been affected. Mendt and collaborators showed that the number and size distribution of sEVs after freezing (for 45 d or 6 months) and thawing are not affected as evaluated by Nanoparticle Tracking Analysis (NanoSight) and electron microscopy compared with freshly prepared sEVs and for up to 48 h when stored at room temperature or 4°C (Mendt et al., 2018). Recently, it has been shown that sEVs should be stored at 4°C or -20°C for short-term preservation; for longer periods of storage for therapeutic application, the most suitable temperature of preservation of sEVs is −80°C (Wu JY. et al., 2021). The effects of storage do not seem to depend on the sEV cellular or tissue source (Maroto et al., 2017).

A correct cryopreservation of biological material intended for therapeutic use is also crucial. When frozen and kept properly, specimens maintain their viability and prevent osmotic damage through the use of cryoprotectants, such as sucrose or trehalose (Kusuma et al., 2018). These cryoprotectants do not penetrate in the cell membrane. Instead, they stabilize and preserve the cell membranes, allowing regulated extracellular ice growth during controlled cooling, thus protecting sEVs from cryodamage (Bosch et al., 2016). There is still a critical need to develop protocols for collection and storage of sEV samples due to the still undefined standard operation procedures for their preservation conditions after isolation. Emerging techniques for EV storage include the lyophilization and freeze-dried sEVs can be stored at −20°C, a technique considered as a cost-saving strategy and holding great promise for translation into therapeutic protocols (Yuan F. et al., 2021). However, such studies are preliminary and require further investigation and standard protocols. More work is needed in this area and the impact on long-term storage and cold chain processes shall be considered.

## 3 Isolation and Characterization of sEVs

MISEV guidelines provide recommendations for the nomenclature, collection and pre-processing, separation and concentration, characterization and functional studies of EVs (Théry et al., 2018). Standardized methods for sEV isolation and analysis are needed to meet the regulatory requirements of their use as drug delivery systems (Rai et al., 2021). Several conventional methods have been employed to isolate sEVs, each one with its own pros and cons, influencing even the yield of sEVs. Table 1 summarizes the advantages and disadvantages of the most common methods for sEV isolation. After sEV isolation, a detailed characterization is required to determine their physicochemical properties, as well as biochemical and molecular composition. There are several techniques to perform such characterization and, usually, more than one method is used to better define the sEV characteristics, as described in this article.

**TABLE 1:** Advantages and disadvantages of most used methods to isolate small extracellular vesicles (sEVs).

Methods	Advantages	Disadvantages	References
Differential ultracentrifugation	Relatively simple and low cost, high purity, high-enrichment isolation, potentially sterile, reduced contamination risks with separation reagents, large sample capacity, scalable	Requires ultracentrifuge machine, laborious and large sample volume, low recovery, protein contamination risks, potential exosomal aggregation, subject to operator-based variability, high speed centrifugation may damage sEVs	(Whitford and Guterstam, 2019; Konoshenko et al. (2018)
Density gradient centrifugation	More specific for certain sEV types, higher separation efficiency, separated components are not mixing again, maintains the structure and functions of sEVs	Complex, time-consuming, considerable sEV loss, expensive	(Lamparski et al., 2002; Zhang et al., 2019a; Lane et al., 2017; Théry et al. (2006)
Ultrafiltration	Sterile, fast, no special equipment required, direct RNA extraction possible, highest exosomal RNA yield, scalable	Low purity, loss of sEVs due to their attaching to the membranes, shear stress that can induce deterioration	(Zhang et al., 2019b; Lane et al., 2017; Oeyen et al., 2018; Heinemann et al. (2014)
SEV precipitation	Simple procedure, low number of steps, preservation of bioactivity, potential high yield, does not require specialized equipment, large and scalable sample capacity	Long running time, co-precipitation of other non-exosomal contaminants (e.g., proteins and polymeric materials)	(Momen-Heravi et al., 2013; Patel et al., 2019; Niu et al. (2017)
Immunological separation	Excellent for isolation of specific sEVs, rapid, easy to use, requires less sample volume, high RNA yield, high sensitivity and specificity, low number of steps	High reagent costs, low yield, difficulty in completely removing antibody from sample	(Théry et al., 2006; Momen-Heravi et al., 2013; Tauro et al., 2012; Cai et al. (2018)
Microfluidics-based technologies	Biocompatibility, requires low sample volumes, high purity, high resolution, contact-free manipulation, low cost, high-throughput, and precision	Low sample capacity, no distinction between the vesicles with the same surface markers	(Guo et al., 2018; Contreras-Naranjo et al., 2017; Sancho-Albero et al. (2020)
Tuneable resistive pulse sensing	High resolution, more accuracy, allows high-throughput analysis and simultaneous evaluation of size and zeta potential	Risk of pores getting clogged, lacks sensitivity, no detection of small sEVs, no distinction between types of particles	(Kurian et al., 2021; Lane et al., 2015; Akers et al., 2016; Coumans et al. (2014)

### 3.1 Traditional and Novel Methods for the Extraction

#### 3.1.1 Differential Ultracentrifugation

Differential ultracentrifugation is the gold standard and the most used isolation method for sEV isolation from body fluids and conditioned media. Although various protocols are available, generally it consists of multiple steps: 1) relative low-speed centrifugation (1,000 g for 10 min), to remove cells and apoptotic debris; 2) higher speed spin (varies among laboratories, from 1,000 g to 20,000 g for 1 h) to eliminate larger vesicles; and 3) a high-speed centrifugation (100,000 g for 2 h) to precipitate sEVs (Zhang P. et al., 2019). Note that biological samples with high viscosity require a longer ultracentrifugation step and higher speed of centrifugation. An additional filtration step is recommended prior to the ultracentrifugation process to remove microparticles, which are selected according to their size (pore diameters of 0.10, 0.22, or 0.45 μm). Importantly, the additional steps in sEV purification (washing and microfiltration) increase the purity of sEVs but also decrease the yield of sEVs that are obtained (Konoshenko et al., 2018; Whitford and Guterstam, 2019). This approach has several advantages, such as easy to use and little technical expertise, but it is also time-consuming. For more purified sEVs and to eliminate contaminations, the pellet can be washed again in a large volume of PBS and centrifuged one last time at 100,000 g for 2 h (Konoshenko et al., 2018). Lately, the isolation of exomeres with a ultra centrifugation at 167,000 g and of supermeres by an additional 367,000 g ultracentrifugation of the exomere supernatant (Clancy et al., 2021) was described. Pellets should be resuspended in PBS and stored at -80°C for further characterization and analysis.

#### 3.1.2 Density Gradient Centrifugation

Density gradient centrifugation is another ultracentrifugation procedure frequently employed. It uses an inert gradient medium for centrifugal sedimentation or sedimentation equilibration (Lane et al., 2017). The sample is placed on a preconstructed density gradient, such as sucrose, iohexol, or iodoxinol, and when a certain centrifugal force is applied, the particles will begin their sedimentation through the gradient in separate zones according to their size, shape density, and the sedimentation coefficient(s) (Théry et al., 2006; Zhang P. et al., 2019). Samples are centrifuged for 16–24 h at 120,000 g at 4°C, and the resulting pellet contains the isolated sEVs collected at their characteristic density zone (1.1–1.2 g/ml) to separate from other components in the sample (Lamparski et al., 2002; Choi et al., 2021). Factors such as centrifugation time, relative centrifuge force, and temperature are particularly important and can lead to inconsistencies in isolated material. Therefore, an adequate centrifugation time avoids the presence of contaminating particles in the sEV fractions due to similar densities (Théry et al., 2006; Lane et al., 2017).

#### 3.1.3 Ultrafiltration

Ultrafiltration is one of the most popular size-based techniques for the isolation of sEVs. The currently available commercial membrane filters have pores of different diameters to allow size distribution, simplifying the process of particle isolation. In some differential centrifugation methods, filtration is used in combination with ultracentrifugation or as an additional step in gel filtration chromatography (Lane et al., 2017; Oeyen et al., 2018). Based on their size, sEVs can be isolated using membrane filters with defined molecular weight or size exclusion limits (Heinemann et al., 2014). During the initial step, the larger vesicles are removed by filters with a pore diameter of 0.80 and 0.45 μm, and the particles with a smaller size than sEVs are separated from the filtrated at the next stage, and a concentrated sEV population is collected. The isolation step requires a relatively short period of time, although the method needs a pre-incubation of the silicon structure with the PBS buffer (Lane et al., 2017). In the following step, the sEV population is concentrated on the filtration membrane. Currently, there are commercial sEV isolation kits available that allow their extraction in shorter periods of time (Hu et al., 2021). Although filtration technologies are faster than ultracentrifugation and do not require special equipment, it is difficult to remove the remaining proteins that adhere to the nanomembrane and hamper the elution of sEVs. Additionally, the use of mechanical pressure may result in the deformation and breaking up of large EVs (Heinemann et al., 2014; Zhang P. et al., 2019).

#### 3.1.4 sEV Precipitation

sEVs can be isolated from biological fluids, by altering their solubility or dispersibility. This method is based on the precipitation of sEVs in solutions of superhydrophilic polymers. One of them is polyethylene glycol (PEG), which has been used to change the sEV membrane surface structure. The use of PEG can help to solve the problem of rapid sEV clearance by phagocytic systems. Indeed, PEG confers more stable properties to sEVs, increasing their time in circulation (Kooijmans et al., 2016), which may be important to improve their efficacy as drug delivery systems. The procedure usually includes mixing the biological fluid with a polymer containing the precipitation solution, incubation at 4°C overnight, and sedimentation of sEVs by low-speed centrifugation (1,500 g). The resulting pellet is then resuspended in PBS for further analysis and sedimentation of sEVs by low-speed centrifugation (1,500 g) to remove cellular debris (Momen-Heravi et al., 2013; Patel et al., 2019). The water-excluding polymers (usually PEG of 8,000 Da) tie up water molecules and exclude less soluble components. sEV precipitation is easy to use, does not require any specialized equipment, has minimal costs, and can be scalable for larger quantities. Moreover, the precipitation with PEG allows us to work in physiological pH ranges and without dependence on the ion concentrations. However, polymer-based sEV precipitation is accompanied by co-precipitation of other non-sEVs contaminants, such as proteins and polymers (Niu et al., 2017; Patel et al., 2019). Currently, several commercial kits using PEG for sEVs isolation are available and easy to use, without the need of additional steps.

#### 3.1.5 Immunological Separation

Immunoaffinity approaches exploit the highly specific affinity interactions between an antigen and an antibody. All the molecules in the surface of sEVs, such as proteins, receptors, lipids, and polysaccharides, are potential ligands. Ideally, sEV biomarkers for immune isolation are highly concentrated or only present on the surface of sEVs and lack free counterparts. Some of the sEV biomarkers (Figure 2B) include tetraspanins, heat shock proteins and MHC antigens, CD9, CD10, CD24, CD63, CD81, EpCAM, Alix, AQP2, FLT1, TSG101, and HSP70 (Tauro et al., 2012; Konoshenko et al., 2018). Several techniques of immunological separation of sEVs have been developed and include antibody-coated magnetic beads, immune-modified superparamagnetic nanoparticles, and microplate-based enzyme-linked immunosorbent assay (ELISA). Antibody-coated magnetic beads and paramagnetic beads coated with antibodies are usually incubated with conditioned culture medium for 24 h at room temperature and sEV complexes are isolated from the magnetic particles with the help of a magnet. Afterward, the obtained sEVs are washed and then assayed, using sEV intracellular proteins as specific markers for their isolation. The diversity of antibodies and fixed phases has given rise to a large number of protocols for the isolation of sEVs, but isolation from larger volumes encounters certain difficulties (Momen-Heravi et al., 2013; Cai et al., 2018). Microplate-based ELISA methods have been developed for capturing and quantifying sEVs from biological samples (plasma, serum, and urine) and the results are expressed as absorbance values relative to the expression of known surface biomarkers. The absorbance values can also be extrapolated to quantify the captured sEVs through calibration using standards with known sEV counts (Théry et al., 2006). It is then possible to characterize and quantify sEV proteins with antibodies against sEV-associated antigens, either common to all sEVs, or specific to sEVs from certain cell types or cell conditions. Following isolation of sEVs, the sub-populations of interest can be separated by differentiating protein markers, avoiding contaminants. The method is applied for detection, analysis, and quantification of both common and cell type-specific sEV proteins (Tauro et al., 2012). The main advantage of the immunological separation is the high purity of the resulting isolated sEVs. Moreover, this approach has a comparable or even greater yield when compared to ultracentrifugation and precipitation-based methods. Nonetheless, this technique has high costs and difficulties related to the detachment of molecules, the analysis of intact vesicles, and the availability of antibodies (Tauro et al., 2012; Momen-Heravi et al., 2013).

#### 3.1.6 Microfluidic-Based Technologies

In the last decade, research efforts have been made to develop microfluidic platforms that offer advantages in combining the separation and detection of sEVs into a single chip (Guo et al., 2018). Microfluidic technologies use small volumes of fluids (nano to microliters) and can be classified as size-based sEV and immunoaffinity-based sEV isolation procedures (Contreras-Naranjo et al., 2017). The system of size-based sEV isolation uses principles of chromatography and has the advantage of obtaining uniformly sized samples (Sancho-Albero et al., 2020). Samples are filtrated through two membranes with a pore size of 20 and 200 nm in diameter and the particles greater than 200 nm remain in the sample chamber (Lin S. et al., 2020).

Dialysis membrane can be used to increase the separation efficiency and purity of samples, or electrophoresis can be employed to force passage of particles across the filter (Contreras-Naranjo et al., 2017). Immunoaffinity-based microfluidic devices are comprised of modified microchannels with antibodies or magnetic beads constructed to capture sEVs based on specific biomarkers, such as CD63, CD81, and the major histocompatibility complex I (MHCI) (Lin S. et al., 2020). In one of the proposed systems, the multiple circular wells are connected through straight channels to increase the interaction with the functionalized surface, with additional narrow channels between the chambers to allow the changes in fluid velocities (Kanwar et al., 2014). Microfluidic-based technologies offer advantages such as high purity even for small quantities of fluids, fast isolation speed, high yield, and low cost in comparison with classical purification methods. Nevertheless, they require expensive and advanced equipment, as well as premixing and incubation of capture beads with samples (Guo et al., 2018; Kurian et al., 2021).

#### 3.1.7 Tunable Resistive Pulse Sensing

Tunable Resistive Pulse Sensing (TRPS) allows high-throughput measurement of individual particles that move through a size-tunable nanopore. TRPS technology, usually applied for a particle size distribution and concentration measurement, has shown promising results as a method for the analysis of sEV samples (Lane et al., 2015). Particles like lEVs and sEVs are detected as a transient change in the ionic current when they move across the size-tunable nanopore, resulting in a resistive pulse signal (Akers et al., 2016). The signal obtained can then be used to calculate the size, charge, and concentration of particles by correlating the fluctuations in the current flow after calibration with a known standard (Kurian et al., 2021). The size of this pore can be adjusted allowing its usage for a variety of samples of different sizes. The technique provides higher resolution, high-throughput analysis, and more accuracy than by light scattering based-techniques (Coumans et al., 2014; Lane et al., 2015). The disadvantages of the TRPS include the lack of sensitivity to sEVs and the risk of pore obstruction with repeated usage (Coumans et al., 2014).

### 3.2 Size and Morphology

Standard flow cytometry is one of the most common and prevalent tools used to analyze the origin, size, and morphology of lEVs, while high resolution flow cytometry and imaging flow cytometers have been used for sEVs, as reviewed in MISEV 2018 guidelines (Théry et al., 2018) and recently published (Botha et al., 2021) Such a high-throughput, multi-parametric technique quantitates thousands of single cells or particles and quickly analyzes both their relative size and granulation (Ko et al., 2016; Tertel et al., 2020; Botha et al., 2021).

The working principle of a flow cytometer is that a laser beam with a specific wavelength is directed through a stream of fluid containing suspended particles and the scattered light is converted to an intensity-associated voltage pulse that can be quantified later. The degree of light scattering depends on the presence of particles in the samples, and sEVs can be quantified and/or classified accordingly to antigen expression levels using specific fluorescently labeled antibodies (Konoshenko et al., 2018). This same approach is also used by some commercially available kits and enables parallel multiple surface biomarker detection with different fluorescent antibodies. However, sEVs are too small to allow that the standard flow cytometry captures the florescence signal, which can be increased through the usage of immunoconjugated beads (Ko et al., 2016). Flow cytometry is a methodology that can be combined with molecular methods increasing statistical robustness and allowing simultaneous analysis of various antigens. Nevertheless, there is still a lack of robust protocols reported, and many studies regarding flow cytometry of sEVs have incomplete methodological descriptions and insufficient calibration and standardization processes to generate the data, as indicated in MISEV guidelines for flow cytometry experiment with EVs (MIFlowCyt-EV) (Welsh et al., 2020). Some studies have used engineered CD63eGFP-labeled sEVs and imaging flow cytometry as a robust multi-parametric detection and quantification of single sEVs and sEV subsets in heterogeneous samples (Görgens et al., 2019). In recent years, ExoView® platform was proposed. It allows the addition of fluorescent antibodies on single sEVs and other EVs. No purification is required, allowing us to quantify the expression of relative proteins with a single fluorescent antibody sensitivity, while simultaneously measuring their size and number (Harkonen et al., 2019). Some of the biophysical approaches routinely used for the characterization of sEVs are focused on measuring the size distribution and morphology. Dynamic light scattering (DLS) allows the assessment of sEV size distribution based on the intensity of the scattered light (Frisken, 2001). Similar to DLS, the nanoparticle tracking analyzer (NTA) uses a laser beam to illuminate sEVs in the sample (Filipe et al., 2010). The suspended sEVs are illuminated by a laser, and the intensity of the light fluctuates over time since sEVs go through Brownian motion, that is, they are then identified and related by movement and particle size based on the Stokes-Einstein equation, where the diffusion velocities are inversely proportional to the size of particles. Both methods provide accurate and sensitive size distributions, but while DLS gives the diameter range of the analyzed vesicles, NTA is capable of tracking a single sEV overcoming polydispersity problems (Frisken, 2001; Filipe et al., 2010). Lately, sEV protein detection has been possible thanks to the quantum dot method coupled with immunomagnetic capture and enrichment. sEVs are captured by magnetic beads based on CD81 protein expression and subsequently detected by fluorescent spectroscopy (Vinduska et al., 2021).

Transmission electron microscopy (TEM) has a higher resolution compared with the other electronic microscopy techniques and has similar procedures for fixation and contrast enhancement. It is widely used to characterize the structure, morphology, and size of various biological components (Figure 1) (Théry et al., 2006). In the scanning electron microscopy (SEM) technique, a focused electron beam causes electron emission from the sEV surface. Samples are chemically or cryogenically fixed followed by dehydration and sputter-coated with a thin layer of gold or carbon for imaging (Chuo et al., 2018). These electrons are collected and magnified using a special lens. Usage of immune-gold labeling in TEM and SEM, as well as cryo-TEM allows the observation of EVs in their hydrated-like native state (Linares et al., 2017).

### 3.3 Biochemical Analysis of sEVs Markers

ExoCarta was previously described in 2009 and is routinely used by researchers to validate and/or characterize their findings in sEVs (Mathivanan and Simpson, 2009). ExoCarta is an online database that allows various groups to establish sEV markers with detailed information about lipids, proteins, and RNA sequences that were identified in specific sEV preparations (Mathivanan et al., 2012). Up to now, approximately 10,000 different proteins and more than 3,000 mRNAs have been characterized (http://www.exocarta.org/). ExoCarta contains annotations on the study that identified the molecule, the sEVs isolated from a particular sample, the employed methods of isolation and identification, and the date of publication. Free web-based resources like Exocarta, and others such as EVpedia and Vesiclepedia, help the scientific community in elucidating the molecular mechanisms and pathophysiological effects of EVs, specifically of sEVs, on target cells, as well as on their therapeutic potential (Mathivanan et al., 2012; Simpson et al., 2012). Recently, the topics on EVs have been updated, providing suggestions on molecular markers for their characterization based on protein composition, as well as on isolation and standardization techniques (Théry et al., 2018).

Western blot is used to separate and identify proteins and can be applied to detect and confirm the presence and expression levels of the sEV-specific proteins in purified sEV samples of tissue homogenate or extracts (He et al., 2014). After sEV isolation, they can be lysed and used for proteomics or be transferred onto a membrane for Western blotting. The specific markers frequently used as sEV markers are the characteristic proteins that belong to their biogenesis process and include, besides those pointed out in Section 3.1.5, Rab GTPase, annexins, flotillin, and the endosomal sorting complexes required for transport (ESCRT) pathway-dependent protein Alix (Simpson et al., 2009). It is a useful technique to identify proteins that are associated with sEVs once it can process multiple proteins at the same time. It is also important to state that although Western blot is often used to detect and confirm the presence of proteins in sEVs, it cannot completely exclude contaminants from different vesicles (He et al., 2014; Zhang P. et al., 2019). An alternative to Western blot, ELISA may be performed, which is a less time-consuming technique that also requires a smaller amount of sample. This technique uses sEV target specific antibodies immobilized to a solid surface to capture sEVs, and is followed by labeling with a detection antibody (Logozzi et al., 2020). Nevertheless, both Western blot and ELISA have some disadvantages, such as low specificity and quality, as well as higher costs.

One of the important characteristics of sEVs is that they may contain nucleic acids (DNAs, RNAs, miRNAs) that will play a crucial role in cell-to-cell communication and gene regulation in the target cell. These nucleic acids can be easily quantified by real-time quantitative reverse transcription polymerase chain reaction (RT-qPCR). This technique has several advantages since it does not need high sample volumes, it has higher sensitivity and resolution capacity, while allows a high-throughput analysis and absolute quantification (Chevillet et al., 2014). RT-qPCR is also important for the characterization of sEVs after transcriptomics. miRNAs have been described either as biomarkers and targets for modulation in several pathologies, including the neurodegenerative diseases (Brites, 2020), which turn these EVs as important tools for disease diagnosis and better understand the disease spread. Recent studies reported other forms of DNA (e.g*.,* genomic and mitochondrial DNA) inside sEVs (Thakur et al., 2014; Sansone et al., 2017), although the exact role of them and the underlying mechanisms are still to be elucidated. Preliminary studies relate mitochondrial DNA as a biomarker of cancer aggressiveness (Arance et al., 2021) and its depletion in sEVs from astrocytes was associated with cell malfunction and spread of pathology (Ha et al., 2021).

## 4 sEVs-Loading With Therapeutic Agents

sEVs show the advantage to have membranes structurally comparable to other membranous structures found in cells in terms of lipid composition. Several proteins including receptors, transcription factors, enzymes, extracellular matrix proteins, lipids, and nucleic acids (DNAs, mRNAs, and miRNAs) are inside and on the surface of sEVs (Mashouri et al., 2019). In terms of lipids, the existence of lipid rafts deserves to be noted (Figure 2B), with ceramides, sphingolipids, and cholesterol, as well as phosphatidylserine and phosphatidylcholine, though to a lesser extent (Skotland et al., 2019). In addition, enzymes, such as phosphatases, proteases, and glycosidases, may also be present in sEVs where they often recapitulate the cell of origin and their metabolic activity (Skotland et al., 2017). sEVs have been referred as being enriched in sphingolipids, cholesterol, and phosphatidylserine in comparison with the donor cells, which may promote a higher stability of these vesicles against detergents than other EVs (Skotland et al., 2019). This means that they have important properties, namely high biocompatibility, enhanced stability, potential targeting, docking modalities, and limited immunogenicity (schematically represented in Figure 3). Cell adhesion molecules (CAMs), such as integrins, tetraspanins, and MHC class I and II, are some examples of specific types of sEV proteins. Also present are the less specific Rab2, Rab7, flotillin, annexin, Alix, heat shock proteins, and cytoskeleton proteins (e.g., actin, myosin, and tubulin) (Mashouri et al., 2019). Together with the MHC class II are integrins involved in antigen presentation and pattern recognition receptors important for innate immunity (Brites and Fernandes, 2015).

All these properties provide potential advantages of sEVs over conventional drug delivery nanocarrier systems and are summarized in Table 2. A variety of therapeutic material, such as drugs, short interfering-RNA (siRNA), antagomirs, pre-miRNAs, and recombinant proteins can be loaded into sEVs during their formation or after their isolation. These approaches may result in different loading efficiencies and stabilities of the drugs in the sEVs**.**

**TABLE 2:** Main differences between conventional nanocarriers and small extracellular vesicles (sEVs) as drug delivery systems.

	Conventional nanocarriers	sEVs
Size	10–300 nm	50–200 nm
Composition	Polymers (natural or artificial; hydrophilic or lipophilic), lipids, silver, polysaccharides	Lipids, proteins, mRNAs, miRNAs, depending on cell of origin
Advantages	Loaded cargo hydrophilic and/or lipophilic drugs, long circulating (PEGylated), manufacturing methods available (liposomes)	Long body circulation, loaded cargo hydrophilic and/or lipophilic drugs, targeted delivery, biocompatibility, safe, biodegradable, cargo protection, immuno-compatibility (homologous), possibility of specific organotropism, high stability
Disadvantages	Immuno-compatibility of some materials, rapid clearance after *in vivo* administration, irregular biodistribution	Low drug loading, diversity of composition depending on cell source, difficult to obtain, long-term effect unclear, possibility of contamination with other EVs

### 4.1 Cell-Based Loading Approach

The therapeutic agents (drugs, proteins, and nucleic acids) can be incorporated into cells that encapsulate the material during the production of sEVs. This strategy is more limited, since it depends on the cellular processes that are responsible for the production of sEVs as well as the cargo loading of the molecules. It also requires that the cellular source (cell line, primary culture) used is compatible and retains viability during the production of sEVs. In this case, after incubation with the therapeutic agents, the donor cells secrete sEVs that will be loaded with both biologically produced components and/or the drug. Such an approach requires that the donor cell can tolerate high concentrations of the drugs in order to be effective (Pessina et al., 2011). As so, it is also difficult to control the loading efficiency of the therapeutic molecule, which may suffer degradation in the host cells. Nevertheless, a major advantage is to specifically target sEVs against disease mechanisms (Garcia-Manrique et al., 2018). Another approach is to transfect the donor cells by genetic engineering with the drug-encoding DNA, which can then be expressed and sorted into sEVs. sEVs full of proteins encoded by the inserted genes can be obtained by isolating and purifying the sEVs through the natural packaging processes. Some disadvantages in this case are that the drug entrance is limited for its encoding DNA yield, depends on the transfection efficiency, and the transfection agents may lead to a reduction of cell viability (Johnsen et al., 2014; Batrakova and Kim, 2015).

### 4.2 Cargo Loading of sEVs Isolated From Donor Cells

The simplest strategy for loading therapeutic agents into sEVs is mixing them with the free drugs, as first demonstrated by Pascucci and others in 2014. Paclitaxel was selectively taken up by MSCs and then incorporated into the released sEVs, at a concentration sufficient to inhibit the growth of tumor cells *in vitro* (Pascucci et al., 2014). The process can also be used to load sEVs with small molecules. For cargo loading after isolation, sEVs are isolated and purified from biological fluids, such as cell culture media, plasma, serum, or milk for the downstream processing. Several methods have been proposed to encapsulate therapeutic agents in sEVs with the common goal of permeating its membrane. These techniques include freeze-thaw cycles, saponin permeabilization, sonication, extrusion, and electroporation procedure. The process of loading should allow the maintenance of the structure and activity of the drugs.

In the method of freeze-thaw cycles, drugs are incubated with sEVs at room temperature for a fixed amount of time, and the mixture is subsequently frozen at −80°C or in liquid nitrogen, and re-thawed at room temperature. This process is repeated for at least three cycles to ensure drug encapsulation. However, the method can induce aggregation of the sEVs, while the drug loading efficiency is generally lower than that of sonication or extrusion methods. sEVs are shown to resist multiple freeze-thaw cycles and are stable when stored from −20°C to −80°C and when subjected to multiple freeze-thaw cycles (Haney et al., 2015; Sato et al., 2016).

Another way to encapsulate drugs in sEVs is to perform a permeabilization with saponin. Saponin, as a natural surfactant, interacts with membrane-bound cholesterol, creating pores that increase the permeability of sEV membrane, thus favoring the encapsulation (Chen et al., 2021). The method appears to not be affected by sEV morphology. However, such an approach is described to have poor stability *in vivo*, as well as low encapsulation yield when saponin is added (Fuhrmann et al., 2015; Haney et al., 2015).

Sonication consists of a method where sEVs derived from donor cells are mixed with drugs or proteins using a sonicator probe. The mechanical shear force from the sonicator probe induces deformation, compromises the membrane integrity of the sEVs, and allows the drug to flow into the sEVs. Nevertheless, in some cases, drugs can also be found in the outer layer of the sEV membrane. There are no significant changes in the structure and content of their membranes after sonication, and the drug sEV formulation has shown to be kept stable under various conditions for over a month (Kim et al., 2016; Liu et al., 2019).

Another method is extrusion. In this method, sEVs from donor cells are mixed with a drug, and the mixture is loaded into a syringe-based lipid extruder and extruded through membranes with 100–400 nm porous size, under a controlled temperature. During the extrusion, the sEV membrane is disrupted and vigorously mixed with the drug, resulting in drug loading into the sEVs. However, the use of this method can lead to changes in EV size, composition, and delivery capacity (Fuhrmann et al., 2015; Haney et al., 2015).

Finally, in electroporation, an electrical field (short, high-voltage pulses) is applied to a suspension of sEVs and the drug cargo of choice, creating small pores in their membrane, thereby facilitating the passage of cargo into the lumen of the sEVs. The integrity of the vesicle membrane is then recovered, resulting in the formation of drug-loaded vesicles. Electroporating a mixture of drug and sEVs at 1000 kV for 5 min was demonstrated to successfully load the drug into the sEVs (Kim et al., 2016; O'Loughlin et al., 2017).

Lately, efficient delivery of siRNAs and miRNAs into sEVs was shown to be achieved with commercial transfection kits, as the Exo-Fect^TM^ from System Biosciences (SBI; System Biosciences, Palo Alto, CA) (Chivero et al., 2020). It was demonstrated to be more efficient than electroporation, heat shock, saponin, or cholesterol-mediated, at least for miRNAs, where more than 1000-fold upregulation was achieved, as compared to native sEVs (de Abreu et al., 2021).

### 4.3 sEV-Mimetic Nanovesicles as Delivery Systems

sEVs are stable and long-circulating endogenous nanocarriers that provide protection of the drug cargo from degradation, while increasing drug delivery to the targeted tissues. One of their major advantages is that they can cross the BBB, thus penetrating into the CNS. On the other hand, some disadvantages may arise from the fact that some components carried by natural sEVs are incompatible with therapeutic purposes, with difficulties in the purification methods and also that cells release relatively low quantities of sEVs (Zhang Y. et al., 2019). In this context, liposomes may be an alternative, as they are small artificial vesicles of spherical shape that can be created from cholesterol and natural non-toxic phospholipids. Due to their size and unique structure, liposomes can compartmentalize and solubilize both hydrophilic and hydrophobic materials. However, liposomes also have some limitations due to their poor stability under shelf and *in vivo* conditions (Akbarzadeh et al., 2013). Modified sEVs with other nanostructures, such as liposomes, may be considered a new outlook of biological drug delivery systems. They are inspired in EVs but represent novel biological nanocarriers. sEV-mimetic nanovesicles contain only the crucial components of natural EVs and are highly biocompatible with efficient targeting ability (Garcia-Manrique et al., 2018). These EVs are obtained by: i) subjecting cells to physical processes producing EVs of nano-dimensions; ii) fusing the membranes of sEVs and liposomes; and iii) coating nanoparticles with a lipid bilayer of cell plasma membranes (Luan et al., 2017; Colao et al., 2018), as detailed in this section.

#### 4.3.1 Cell Membrane-Derived EVs as Bioactive Nanocarriers

Cell membrane-derived EVs are the natural analogs of liposomes. They can be fashioned from larger membrane structures, commonly prepared by size extrusion of monocytes or macrophages (Whitford and Guterstam, 2019). The forceful and sequential passage of cells through polycarbonate membrane filters (10, 5, and 1 μm pore sizes) leads to the generation of a large amount of nanovesicles, with a final size between 50 and 200 nm. Nanovesicles can also be obtained through the passage of cells over hydrophilic microchannels, generating a delivery system for endogenous material with technical features similar to those of sEVs (Jang et al., 2013; Lunavat et al., 2016). The production of nanovesicles can reach a high yield of cell-derived vesicles from living cells over a relatively brief period of time, achieving until 100 times more yield in comparison with the production of sEVs by using the same number of cells. Encapsulation efficiency is dependent on the initial amount of the added drug (Jang et al., 2013).

#### 4.3.2 Engineering sEVs by Fusion With Liposomes as Hybrid Nanocarriers

Hybrid systems produced after fusion between liposomes and sEVs are an attractive opportunity that, in principle, may decrease the immunogenicity of liposomes and increase their colloidal stability, while improving the half-life of the system in the blood. There are two approaches: i) drug-loaded liposomes are incubated with donor cells to produce sEVs or ii) sEVs are treated with drug-loaded liposomes composed of fusogenic lipids (Sato et al., 2016; Garcia-Manrique et al., 2018). In both strategies, features of sEVs depend on the properties of the liposome preparations used for the incubation processes, as well as cell uptake capabilities that vary with the cell type. When compared to neutral or anionic liposomes, cationic liposomes showed different uptake propensities and higher encapsulation than both cationic liposomes and/or non-fusogenic sEVs. Engineered hybrid sEVs seem to improve cellular delivery efficiency of therapeutic agents, as compared to the free drug or to the drug-loaded liposome precursor. Moreover, liposomes coated with peptides or antibodies as targeting moieties or PEG can be used to modify properties of the sEV surface. Furthermore, this approach may promote an efficient loading of larger molecules, mixing the cargo of the synthetic vesicles with that of their natural equivalent, while preserving their intrinsic content and biological properties. Generally, the fusion of cells/sEVs with liposomes increases sEV yield by conventional separation methods (Sato et al., 2016; Luan et al., 2017; Yamashita et al., 2018).

#### 4.3.3 Cell Membrane-Coated Nanoparticles as Nanomedical Tools

Nanoparticles coated by cell membranes mimic the properties of the source cells from which their membrane is derived, providing the ability to synthetic core structures to carry therapeutic cargo. They consist of a synthetic nanoparticle core covered by a natural cell membrane layer, with the advantages of immune-compatibility, long circulation, and disease-relevant targeting (Jang et al., 2013; Whitford and Guterstam, 2019). Recently, many types of membranes, such as those from erythrocytes, immune cells, platelets, stem cells, endothelial cells, activated fibroblast cells, cancer cells, and even *E. coli*, have been developed as carriers to facilitate the undetected targeted delivery of core nanoparticles independently of their properties. Natural cell membranes contain a series of functional moieties (proteins, antigens, and carbohydrates) that participate in protection, specific recognition, and intracellular communication, facilitating appropriate nanoparticle delivery (Fan et al., 2018; Fang et al., 2018; Luchini and Vitiello, 2019). The conventional approaches for production of cell membrane-coated nanoparticles involves three steps: i) membrane extraction from source cells; ii) inner core nanocarrier production; and iii) fusion process of the membranes with nanoparticulate cores (Fang et al., 2018). The cell membrane extraction requires large volumes of cells and includes membrane lysis and membrane purification, which should be as gentle as possible. This process is determined by the cell type of interest and includes freeze-thaw cycling, electroporation, and osmosis-based lysis coupled with physical homogenization. A variety of materials (organic or inorganic) may be utilized to produce cell membrane-coated nanoparticles, but the main criterion is that the nanoparticles have a negative zeta potential, which will facilitate orientation of the membrane around the nanoparticle. After extraction of the cell membrane and the introduction of the inner core nanocarrier, these two materials need to be fused together, without drug loss or protein denaturation (Zhai et al., 2017; Fang et al., 2018). Membrane extrusion, ultrasonic fusion, or electroporation are frequently used. However, for the success of these strategies, optimizations are needed in the voltage, duration, and flow velocity, as well as in the cell membrane-to-nanoparticles ratio, which should be carefully controlled to ensure complete surface coverage with the cell membrane (Fan et al., 2018).

## 5 sEVs as Drug Delivery Systems for Neuroregeneration

### 5.1 Migration Across the Blood-Brain Barrier

Significant efforts have been made to deliver small therapeutic molecules/drugs and diagnostic agents into the brain. The discovery of different types of EV cargo and their ability to interact and be taken up by specific cells has led investigators to concentrate on the potential of sEVs as delivery vehicles for therapeutic applications (Luan et al., 2017; Yamashita et al., 2018). sEVs have an efficient capability to cross the BBB, to deliver their intact content into a specific target cell, while also remain stable in the peripheral circulation, thus contributing to be considered potential attractive nanocarriers in the treatment of several CNS diseases (Alvarez-Erviti et al., 2011; Kojima et al., 2018; Busatto et al., 2021). A study carried out in 2016 reported the transfer of a tight junction protein from the endothelial cells to the leukocytes through EVs, thus supporting their cell-to-cell migration (Paul et al., 2016). In another work using rats with a fluorescently tagged protein expressed selectively in the brain tissue, the authors recovered the labeled sEVs in the blood of those animals and demonstrated that sEVs crossed the BBB in a bi-directional manner (Gomez-Molina et al., 2019). Chen et al. (2016) also showed that sEVs are internalized by brain microvascular endothelial cells through endocytosis, by using confocal microscopy and co-localizing sEVs with endosomes (Chen et al., 2016). In addition, sEVs are internalized by target cells through a variety of endocytic pathways. In particular, they use endogenous receptors that are highly expressed at the surface of the BBB, such as transferrin and insulin receptors, confirming sEVs as having a key role in intercellular communications and passage through the BBB in both directions (Yamashita et al., 2018). However, little is still known about the mechanistic details of sEV migration across the BBB and further investigation is required.

### 5.2 sEVs as miRNA Carriers

The content of sEVs can reflect the cell of origin and depends on the physiological or pathological conditions during their formation, suggesting horizontal transfer of genetic information (Zhang Y. et al., 2019). However, sEVs may also be enriched with a particular set of proteins and microRNAs (miRNAs), through selective mechanisms of protein cargo sorting controlled by specific post-translational modifications (PTMs) (van Niel et al., 2006). miRNAs are short nucleotide sequences of non-coding RNAs and can regulate gene expression in various cell types, constituting a critical factor in intracellular communication between origin and target cells (Simpson et al., 2009; Hessvik and Llorente, 2018; Zhang Y. et al., 2019). In the nervous system, a range of cell types have been shown to release miRNA-containing sEVs, including Schwann cells, microglia, oligodendrocytes, astrocytes, and neurons. For instance, miR-21, usually implicated in the microglial anti-inflammatory response, is transferred from neurons to microglia in a process that is mediated by sEVs (Fernandes et al., 2018). On the other hand, the most abundant miRNA of the CNS, the miR-124, is released from neurons as an sEV cargo and is taken up by astrocytes (Morel et al., 2013). Another study proposed that the delivery of miR-155 from microglia to adjacent cells may be mediated by sEVs (Cunha et al., 2016). In this study, the authors found that the expression profile of inflammation-associated miRNAs in sEVs recapitulated those in the cells on exposure to lipopolysaccharide (LPS), a pro-inflammatory stimulus. Increased expression of miR-21 released by Schwann cells in sEVs constitutes an important feature of the repair program of these cells, contributing to axonal regeneration and functional recovery after nerve injury (Lopez-Leal et al., 2020). miRNA profiles identified in sEVs of the CSF/blood in neurodegenerative disorders reinforce the important role of specific sEVs-miRNAs in the regulation of the neuroinflammatory processes (Ciregia et al., 2017; Yoo et al., 2018). In particular, we recently showed that increased levels of miR-124 in neurons derived from induced pluripotent cells generated from AD patients reduces amyloid precursor protein (APP) gene expression, tau hyperphosphorylation, and prevents dendritic spine deterioration (Garcia et al., 2021). In contrast, upregulation of miR-124 in SOD1G93A mutant motor neurons as a model of amyotrophic lateral sclerosis (ALS) evidenced to associate with neurodegeneration and homeostatic cell imbalance (Vaz et al., 2021). Therefore, miRNA sorting in sEVs may either work as a protective mechanism or be a sign of injury to neighboring cells and is dictated by the cell’s own metabolism. Therefore, these sEVs-containing miRNAs are cell specific and may be involved in many biological and pathological processes, as further detailed in the next section. All these factors should be considered when choosing the cell source of sEVs to be used as therapeutic carriers.

### 5.3 sEVs as Delivery Systems of Therapeutic Agents in Brain Pathology

To date, cell-derived EVs provide multiple advantages over traditional synthetic delivery vehicles and potential new drug delivery methods, such as limited immunogenicity, natural composition, small size (nanoscale), enhanced stability in circulation, slightly negative zeta potential for long circulation, and deformable cytoskeleton. As drug delivery carriers, sEVs have the ability to cross many biological barriers, such as the BBB (Elliott and He, 2021; Yamashita et al., 2018). The main advantages of sEVs to be used as drug delivery systems in neurological diseases were addressed in previous sections and are indicated in Figure 3. To note, however, that the low residence time of sEVs in the circulation because of their rapid clearance by phagocytosis may compromise their efficiency and the generation of invisible sEVs has been attempted (Parada et al., 2021). We next will summarize the application that sEVs may have to prevent, halt, or even regenerate neural cell malfunction in age-associated neurodegenerative diseases and brain tumors.

#### 5.3.1 Alzheimer’s Disease

AD is the most common form of dementia, affecting brain regions that exhibit high synaptic activity implicated in important brain functions, such as memory and learning. sEV content seems to be specific for a particular activation or disease state. It was shown, for example, that sEV levels of both phosphorylated tau and amyloid beta (Aβ) proteins detected in the blood of AD patients were significantly higher than in controls, from 1 to 10 years before disease diagnosis (Fiandaca et al., 2015). Due to the complex pathophysiological process of this disease, its treatment is restricted to a few conventional oral medications that act only superficially (Jaul and Barron, 2017). In the incessant search for new therapeutic strategies, sEVs showed up as innovative approaches to be adopted in AD treatment. Alvarez-Erviti and others (2011) were the first to demonstrate that systemically injected sEVs could cross the BBB. They expressed a fusion protein of Lamp2b and rabies virus glycoprotein (RVG) in dendritic cells, which was incorporated into sEVs. Purified sEVs were loaded with exogenous GAPDH siRNA against BACE1 by electroporation and injected intravenously into mice. Systemic injection of RVG-targeted sEV loaded with GADPH siRNA induced specific gene knockdown in neurons, microglia, and oligodendrocytes and a 60% decrease of BACE1 mRNA in the brain cortex 3 d after administration. Besides the application of the sEVs in gene silencing strategies targeting the brain, also provided insights for their utilization in other tissues, as well.

Another study reported that sEVs, isolated from the serum of thr5xFAD mouse model and patients, when injected in the wild type mice brain are taken up by neurons, shuttle Aβ into the cells, and cause cell death by apoptosis, suggesting that disruption of Aβ at the sEV surface may protect from its induced neurotoxicity (Elsherbini et al., 2020). In contrast, sEVs, isolated from the adipose tissue-derived MSCs, decreased the levels of Aβ in an *in vitro* AD model by increasing the levels of neprilysin, important for Aβ degradation (Katsuda et al., 2013). Another compound that has been studied for many years for its beneficial role in AD is curcumin. Curcumin has anti-inflammatory, anti-lipidemic, and anti-oxidative properties. Due to these effects, it is suggested that curcumin can reduce tau protein clumping in the brain, slowing cognitive deterioration in patients with AD. Curcumin has been shown to improve AD-like symptoms in mice (Strimpakos and Sharma, 2008). Despite these advantages, curcumin exhibits low bioavailability because of its poor solubility, low permeability, and absorption, as well as a faster metabolism rate. Nanoformulations of curcumin in sEVs may overcome such limitations, thus potentiating its usage in the treatment of neurodegenerative diseases. Sun and collaborators (Sun et al., 2010) were the first to demonstrate that sEVs loaded with curcumin increase the bioavailability and stability of the compound *in vivo*. Inspired by all these properties, Wang et al. ((2019) produced sEVs as a specifically designed carrier able to carry curcumin by injection in the right ventral hippocampus of an induced AD mouse model. They used okadaic acid to induce tau hyperphosphorylation and sEVs-enriched in curcumin to prevent neuronal death. In this study, they fabricated sEVs secreted by curcumin-treated mouse macrophage cells (curcumin-primed sEVs) to improve their solubility, stability, and tissue bioavailability. Compared with non-encapsulated curcumin, curcumin-primed sEVs enhanced the stability, and bioavailability of curcumin, inhibiting tau phosphorylation mediated by the AKT/GSK-3β pathway, thus contributing to the effective amelioration of learning and memory deficiencies in the induced-AD mice. Administration of curcumin loaded sEVs isolated from mESCs and administered by alternate nostrils in ischemia injured mice reduced post-ischemic events and restored the neurovascular unit, while the fluorescent sEVs were identified in the brain cortical region (Kalani et al., 2016). Nevertheless, additional *in vivo* studies are needed to better understand the specificity of the curcumin-primed-sEVs for other specific brain areas.

Quercetin is a natural bioactive flavonoid with significant pharmacological effects that has been lately widely studied for its antioxidant biological properties connected to its antioxidant activity (Albarracin et al., 2012; Song X. et al., 2020), and with benefits to counteract the neurodegenerative processes. Recently, it was shown that quercetin is able to inhibit tau hyperphosphorylation by reducing the formation of insoluble neurofibrillary tangles associated with AD (Calfio et al., 2020). In another study, plasma sEVs were chosen as therapeutic cargo carriers, improving the solubility and the brain targeting of quercetin in an animal model of AD (Qi et al., 2020). Quercetin-loaded sEVs were enriched with heat shock protein 70 (HSP70) to improve the BBB crossing by specific active targeting between sEV carrying HSP70 and endothelial Toll-like receptor 4 (TLR4) in the brain. Compared to free quercetin, plasma sEVs loaded with quercetin improved the drug bioavailability in AD and further relieved the symptoms of AD in this model by inhibiting the cyclin-dependent kinase 5-mediated phosphorylation of tau and reducing formation of insoluble neurofibrillary tangles, suggesting its potential therapeutic benefit in AD.

#### 5.3.2 Parkinson’s Disease

PD is a brain disorder characterized by the degeneration of nigrostriatal dopaminergic neurons, as well as by the presence of Lewy bodies resulting from misfolded α-synuclein in the surviving neurons in the striatum. Levodopa, a prodrug of dopamine, remains as one of the main drugs in the treatment of PD because dopamine cannot cross the BBB (Banks, 2016). An alternative strategy adopted by Qu et al. (2018) was to combine sEVs and dopamine, enabling the permeation of dopamine through the BBB with maximum biocompatibility and minimum toxicity. The authors isolated sEVs from human blood and loaded sEVs with a saturated solution of dopamine (24 h at room temperature) and tested the uptake of the loaded sEVs by mouse brain endothelial cells *in vitro* and the targeting ability of labeled sEVs *in vivo*. In both experiments, the authors observed that blood sEVs crossed the BBB and delivered dopamine into the brain through an interaction between transferrin and its receptor. Loaded blood sEVs demonstrated a 15-fold improvement in dopamine brain distribution and showed less toxicity than free dopamine by intravenously systemic administration. The sEVs loaded with dopamine had a continuous dopaminergic stimulation and a more stable treatment effect, with better therapeutic effect in the PD mouse model, demonstrating that this strategy seems to be efficient for obtaining a regular distribution of molecules in the brain (Qu et al., 2018). In another study, it was found that sEVs facilitated the entrance of catalase into the brain parenchyma of PD mice by intranasal administration, which produced a potent neuroprotective effect (Haney et al., 2015). In this study, researchers compared several active cargo-loading techniques for the loading of catalase into RAW264.7 macrophage-derived sEVs. The approach used by Haney and others (2015) focused on direct manipulations of sEVs and their membranes to obtain a more effective drug loading. Another strategy may be the usage of cellular modifications to originate sEVs with increased targeting ability. Indeed, a previous study from the same research group demonstrated that systemic administration of macrophages that were genetically modified to overexpress catalase in the released sEVs with incorporated DNA, mRNA, transcription factors, and the encoded proteins. This resulted in the sustained catalase expression by these macrophages and subsequent potent anti-inflammatory and neuroprotective outcomes in a mouse model of PD (Haney et al., 2013). In both approaches, the catalase was the therapeutic agent delivered by sEVs. Catalase is one of the most important antioxidant enzymes that mitigate oxidative stress to a considerable extent by destroying cellular hydrogen peroxide to produce water and oxygen. Deficiency or malfunction of catalase is postulated to be related with the pathogenesis of many age-associated degenerative diseases among which is PD, though the therapeutic delivery of this protein to the brain is restricted by the BBB (Jaul and Barron, 2017). Making use of a set of sEV transfer into cells, devices enabled efficient and customizable production of designer sEVs in engineered mammalian cells (endogenous modification-based approach). Kojima et al. (2018) also confirmed the functionality of these engineered sEVs to deliver therapeutic catalase mRNA for PD treatment. These genetically encoded devices in sEV donor cells optimized the therapeutic sEV release and controlled the delivery of intended biomolecules, without the need to concentrate sEVs. More importantly, engineered donar cells implanted in living mice allowed for a consistent delivery of mRNA cargo into the brain. The catalase mRNA via sEVs from implanted donor cells attenuated neurotoxicity and neuroinflammation in *in vitro* and *in vivo* models of PD, opening new therapeutic opportunities by enabling the delivery of therapeutic mRNAs *in vivo*. Moreover, recent evidence supports the benefits of blood-derived sEVs from healthy volunteers when intraperitoneally injected in the MPTP-treated C57BL/6 mice, as a model of PD, for neuroprotection, as well as counteract inflammatory pathogenic features and recover motor ability (Sun et al., 2020).

#### 5.3.3 Amyotrophic Lateral Sclerosis Disease

ALS is a progressive and fatal disease that affects motor neurons and glial cells in the brain and the spinal cord. Although most cases are sporadic, aggregation of mutated superoxide dismutase 1 (mSOD1) is a pathological hallmark of a subset of familial ALS. Propagation of mSOD1 may occur though the solubilized protein or by cell-derived sEVs (Grad et al., 2014). However, it was also proposed that sEVs from adipose-derived stem cells (ADSC) of healthy humans promote tissue repair in neuronal stem cells isolated from the subventricular zone of mSOD1 mice carrying the G93A mutation due to their strong immunosuppressive and regenerative effects through the release of paracrine factors (Lee et al., 2016). In this study, the authors proposed that ADSC-derived sEVs are effective in treating cellular phenotypes of ALS, including SOD1 aggregation and mitochondrial dysfunction. Based on these facts, the use of stem cells-derived sEVs may provide considerable advantages over their source cells, with better safety and making them attractive therapeutic strategies in neurodegenerative diseases. Indeed, a study performed in mSOD1 mice showed that systemic injection of adipose-derived MSCs delayed motor deterioration for 4–6 weeks and pointed the up-regulation of glial-derived neurotrophic factor (GDNF) and basic fibroblast growth factor (bFGF) produced by these MSCs via paracrine signaling as candidates for such benefits (Marconi et al., 2013). It was also shown that the incubation of sEVs derived from adipose-stromal cells in ALS motor neurons (NSC-34 cell line) has a neuroprotective effect following an oxidative insult, supporting the idea that sEVs mimic or are even better than neuroprotection by stem cells (Bonafede et al., 2016). A recent *in vivo* study from the same group tested the effects of sEVs isolated from ADSC on the mSOD1 mice and compared the intravenous and intranasal routes of administration (Bonafede et al., 2020). They found that treated mSOD1 mice with either intravenous or intranasal repeated administrations presented an improved motor performance, less cell death of lumbar motor neurons, and lower glial activation, as well as an improved neuromuscular junction functionality and muscle fiber morphology. Interestingly, through magnetic resonance imaging, the authors found that labeled ASC-sEVs reached the CNS administered by the intranasal route and accumulated in the typical lesioned sites of the mSOD1 mice brain, making this a promising tool for effective drug delivery into the CNS.

In another study using ALS NSC-34-motor neurons in a co-culture system with microglia, it was noticed that microglial cells were the main recipients of sEVs derived from ALS motor neurons, and that these sEVs induced phenotypic microglial alterations (Pinto et al., 2017). Since these sEVs are enriched in miR-124, it is conceivable that the modulation of this miRNA may have potential benefits to halt microglia activation and associated effects in motor neuron degeneration in this pathology. Indeed, we recently reported that miR-124 normalization in mSOD1 motor neurons prevented their dysregulation in terms of neurite network, and mitochondria and synaptic dynamics (Vaz et al., 2021). Importantly, this study demonstrated that the secretome (including sEVs), derived from mSOD1 motor neurons modulated with anti-miR-124 was able to counteract the pathology observed in the spinal organotypic cultures from mSOD1 mice in the early symptomatic stage. Such work highlighted miR-124 (either in cells, secretome, and/or sEVs) as a new therapeutic target to be considered in ALS. On the other hand, transfection with pre-miR-146a in astrocytes from mSOD1 cortical brain of mice pups abrogated the aberrant markers described for this cellular population (Gomes et al., 2019; Barbosa et al., 2021). Interestingly, this cellular transfection also counteracted miR-146a depletion in sEVs and led to secretome-mediated miR-146a enhancement in motor neurons and microglia, while recovered their function, reinforcing the miRNA modulation and consequent production of a more neuroprotective secretome/sEVs as emerging therapeutic targets in ALS.

#### 5.3.4 Brain Tumors

sEVs derived from brain endothelial cells were used to deliver chemotherapeutics such as paclitaxel and doxorubicin across the BBB (Yang et al., 2015). In this study, four types of sEVs derived from different cells were isolated from the culture media (glioblastoma astrocytoma U-87 MG, endothelial bEND.3, neuroectodermal tumor PFSK-1, and glioblastoma A-172) and drugs such as rhodamine 123, paclitaxel, or doxorubicin were added to sEVs in PBS and incubated at 37°C for 2 h. The ability of sEVs to deliver drugs that crossed the BBB was examined *in vivo* by injecting bEND.3-derived sEVs loaded with the three molecules in the zebrafish model. Studies of drug biodistribution showed that when delivered by bEND.3 sEVs, there was a significant penetration of the fluorescent marker and of the two anticancer drugs into the brain region of the zebrafish embryos, reinforcing the ability of sEVs to cross the BBB. In the brain cancer model, sEVs with anticancer drugs significantly decreased fluorescent intensity of xenotransplant cancer cells and of the tumor growth marker vascular endothelial growth factor (VEGF) (Yang et al., 2015). The data showed significant therapeutic efficacy in the zebrafish brain model treated with sEVs with doxorubicin compared to doxorubicin alone, showing the potential of sEVs to deliver both small and big molecule drugs across the BBB for the treatment of brain cancers. In addition, an *in vivo* study using genetically engineered EVs carrying the suicide gene for cytosine deaminase (CD) fused to uracil phosphoribosyltransferase (UPRT) demonstrated a reduction in the tumor growth in the glioblastoma mice model after treatment with the CD-UPRT-enriched EVs (Erkan et al., 2017). Zhuang et al. (2011) tested sEVs loaded with the signal transducer and activator of transcription 3 (STAT3) inhibitor and evaluated its effect on brain tumor-bearing mice model. In this model, mice treated intranasally with these loaded sEVs presented enhanced tumor apoptosis and significantly delayed GL26 tumor model, leading to increased survival. Another study observed that sEVs derived from marrow stromal cells were able to deliver anti-tumor miR-146b in an *in vivo* rodent model of malignant glioma, with consequent significant reduction of the tumor volume (Katakowski et al., 2013). Lately, Zhu and others (2019) used sEVs derived from embryonic stem cells (ESCs), which are known to have anti-tumor properties, and showed that they inhibited glioblastoma growth both *in vitro* and *in vivo*. The ESCs-sEVs modified with a targeting ligand for cancer chemotherapy at their surface facilitated their ability to cross the BBB (Zhu et al., 2019). In the same study, the authors also used paclitaxel (a mitotic inhibitor to tumor cells), loaded in the sEVs, and demonstrated that this strategy significantly improved the therapeutic effects of paclitaxel in glioblastoma enhancing mice survival. These results suggest that sEVs derived from ESCs are powerful therapeutic vehicles for glioblastoma treatment.

#### 5.3.5 Other Brain-Associated Diseases

As indicated in Section 5.3.1, curcumin displays a variety of pharmacologic properties, attributed to its antioxidant and anti-inflammatory effects. Curcumin administration by nanocarriers improves several clinically relevant parameters, and considerably increases the chemical stability of curcumin by preventing its enzymatic and pH degradation (Loch-Neckel et al., 2015). Strategies to exploit sEVs and curcumin for the treatment of brain diseases have also been reported. Zhuang et al. (2011) prepared curcumin-loaded sEVs (5 min at 22°C) and evaluated *in vivo* effects in different disease models, namely in the LPS-induced brain inflammation model, and in the experimental autoimmune encephalitis (EAE) after intranasal administration. The effects of the administration of curcumin-loaded sEVs showed significant anti-inflammatory effects and blockade of LPS-induced brain inflammation, as well as of the reduction of myelin oligodendrocyte glycoprotein (MOG) in the EAE model. In these models a concomitant reduction in disease progression was observed, suggesting that intranasal delivery of anti-inflammatory agents provides a promising non-invasive approach for the treatment of brain inflammatory-related diseases. The combination of curcumin and the potentials of sEVs were also adopted by Kalani et al. (2016), aiming at neurovascular restoration following ischemia-reperfusion injury. Curcumin was loaded into sEVs in a proportion of 1:4 and the method of nanopreparation was the rapid freeze-thawing. To determine the therapeutic efficacy of curcumin-loaded sEVs, the authors evaluated the neurological score, lesion volume, and cerebral edema in ischemia reperfusion-injured mice. These results showed that treatment with sEVs isolated from mouse embryogenic stem cells were effective in reducing infarct volume, edema, inflammation, and astrogliosis, while also restored NeuN positive neurons. The intranasal administration for 7 d was able to protect the tight junctions from dysfunction and BBB from disruption following injury by ischemia reperfusion injury in mice. Lately, sEVs from bone MSCs administered by the retro-orbital route into the C57BL/6 mice after traumatic brain injury were shown to modulate microglia/macrophage polarization and ameliorate early inflammatory responses (Ni et al., 2019). To highlight that sEVs isolated from the serum of 3-month-old mice (young) and intravenously injected in the 18-month-old recipient mice (old) reversed the expression of aging biomarkers in the lungs and the livers, but one can expect the same effect in the brain (Lee et al., 2018). All these results demonstrate that sEVs can effectively reach the CNS through different administration routes and are a novel and promising therapeutic approach for brain disorders (Kalani et al., 2016).

## 6 Conclusion and Final Considerations

In recent years, sEVs were proposed as a powerful therapeutic tool that may be used to target brain pathology since they present numerous advantages as therapeutic nanocarriers, as schematically represented in Figure 3. Considering their biocompatibility and ability as efficient cell-to-cell messengers, sEVs may be delivered by various administration routes, to overcome biological barriers and reach the CNS. sEVs have low immunogenicity and are more stable in the circulation than other nanoparticle systems due to their endogenous origin and special surface composition. Moreover, they have the capability of loading multiple molecules, such as drugs, mRNAs, miRNAs, and proteins, allowing a more efficient delivery into specific cells or tissues, by properly modifying their surface based on the parental cell status and/or the source of donor cells. By doing that, their usage avoids most of the drugs being delivered to accumulate in other sites than the intended target. Lately, sEVs can be manipulated to be loaded with specific therapeutic molecules and miRNAs, through the generation of sEV-mimetics or by manipulation of their donor cells. Notwithstanding all the positive evidence to use sEVs as nanocarriers, some aspects still need to be further elucidated, such as the biodistribution analysis of sEVs, the mechanism of the brain entrance through the BBB, and their stability and pharmacokinetic properties. A critical issue that must be considered is the choice of sEV donor cells. Additional information on the complex molecular constitution of sEVs, i.e., their cargo dependence on their cell source, among others, will be imperative for the choice of the therapeutic goal and their usage. In addition, further studies are still required to explore the effects of sEVs *in vivo*, in terms of safety, effective delivery, and avoidance of off-target effects. A better knowledge into the sEV cargo and their cell/tissue specific targets will certainly provide new therapeutic strategies to overcome CNS disorders.
